# Bullöses Wells‐Syndrom erfolgreich mit Omalizumab behandelt

**DOI:** 10.1111/ddg.15964_g

**Published:** 2026-05-05

**Authors:** Giulia Ciccarese, William Andrew Rosato, Francesco Drago, Alexandre Raphael Meduri, Francesca Ambrogio, Aurora De Marco, Gerardo Cazzato, Caterina Foti

**Affiliations:** ^1^ Dermatology Unit Department of Medical and Surgical Sciences University of Foggia Foggia Italy; ^2^ Section of Dermatology Department of Precision and Regenerative Medicine and Jonian Area (DiMePRe‐J) University “Aldo Moro” of Bari Bari Italy; ^3^ Casa di Cura Villa Montallegro Genoa Italy; ^4^ Section of Molecular Pathology Department of Precision and Regenerative Medicine and Ionian Area (DiMePRe‐J) University “Aldo Moro” of Bari Bari Italy

Sehr geehrte Herausgeber,

Das Wells‐Syndrom (WS) ist eine seltene Hauterkrankung mit unbekannter Ätiologie. Aufgrund seiner Ähnlichkeit mit Erysipelen (bakterieller Zellulitis) und einer Blut‐ und Gewebe‐Eosinophilie ist es als eosinophile Zellulitis bekannt. Die klinischen Manifestationen des WS sind heterogen und das histologische Erscheinungsbild ist nicht spezifisch.^1^, ^2^ Da der Krankheitsverlauf chronisch‐remittierend ist, ist in der Regel eine Behandlung erforderlich.^1^, ^2^

Hier beschreiben wir den Fall einer Frau mit der bullösen Variante des WS (BWS), die erfolgreich mit einem monoklonalen Anti‐IgE‐Antikörper behandelt wurde.

Eine 36‐jährige Frau stellte sich mit einem seit 3 Jahren bestehenden juckenden Hautausschlag vor, der aus erythematösen Papeln und urtikariellen Plaques am Rumpf und an den Extremitäten bestand (Abbildung 1a), teils in anulärer Ausprägung (Abbildung 1b). Bei der Patientin wurde eine chronische spontane Urtikaria (CSU) diagnostiziert und sie wurde systemisch mit Antihistaminika und Prednison behandelt (Anfangsdosis 0,5 mg/kg Körpergewicht [KG]/Tag), was zu klinischer Besserung führte. Nach Absetzen der Steroide kam es jedoch zum Rezidiv. Im letzten Jahr waren die Hautausschläge durch juckende Bläschen gekennzeichnet, die zu Blasen an den Knöcheln konfluierten (Abbildung 1c). Die Patientin verneinte die Einnahme von Medikamenten oder Insektenstiche.

**ABBILDUNG 1:**
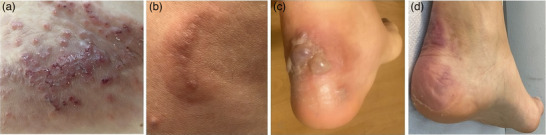
(a) Exkoriierte Papeln, die zu einer Plaque auf dem Rücken der Patientin konfluieren. (b) Erythematöse, bogenförmige Urtikaria‐Plaque am Rumpf. (c) Blasen mit serösem Inhalt am linken Knöchel der Patientin. (d) Vollständige Rückbildung der bullösen Läsionen am Knöchel.

Laboruntersuchungen ergaben eine Eosinophilie im Blut (1,77 × 10^3^/µl; Normalbereich 0–0,53) und erhöhte Gesamt‐IgE‐Werte (338 IU/ml; Normalwert < 100). Das vollständige Blutbild, die Elektrolyte, das C‐reaktive Protein, die Leber‐ und Nierenfunktionstests sowie die Komplementproteine C3 und C4 lagen innerhalb der Normwerte. Antinukleäre und anti‐extrahierbare nukleäre Antigen‐Antikörper waren negativ, ebenso wie Autoantikörper gegen das 180‐kD‐*Bullous‐Pemphigoid* (BP)‐Antigen (BP180), das 230‐kd‐BP‐Antigen, Desmoglein (Dsg)‐1, Dsg3 und Kollagen VII. Die parasitologische Untersuchung von drei Stuhlproben und die Ultraschalluntersuchung des Abdomens lagen innerhalb der Normwerte.

Die Histologie einer bullösen Läsion zeigte Nekrose der Keratinozyten, eosinophile Spongiose und ein diffuses eosinophiles Infiltrat in der Dermis in Verbindung mit einigen Flammenfiguren (Abbildung 2a–c). Die direkte Immunfluoreszenz zeigte keine Ablagerung von Immunglobulin oder Komplement.

**ABBILDUNG 2:**
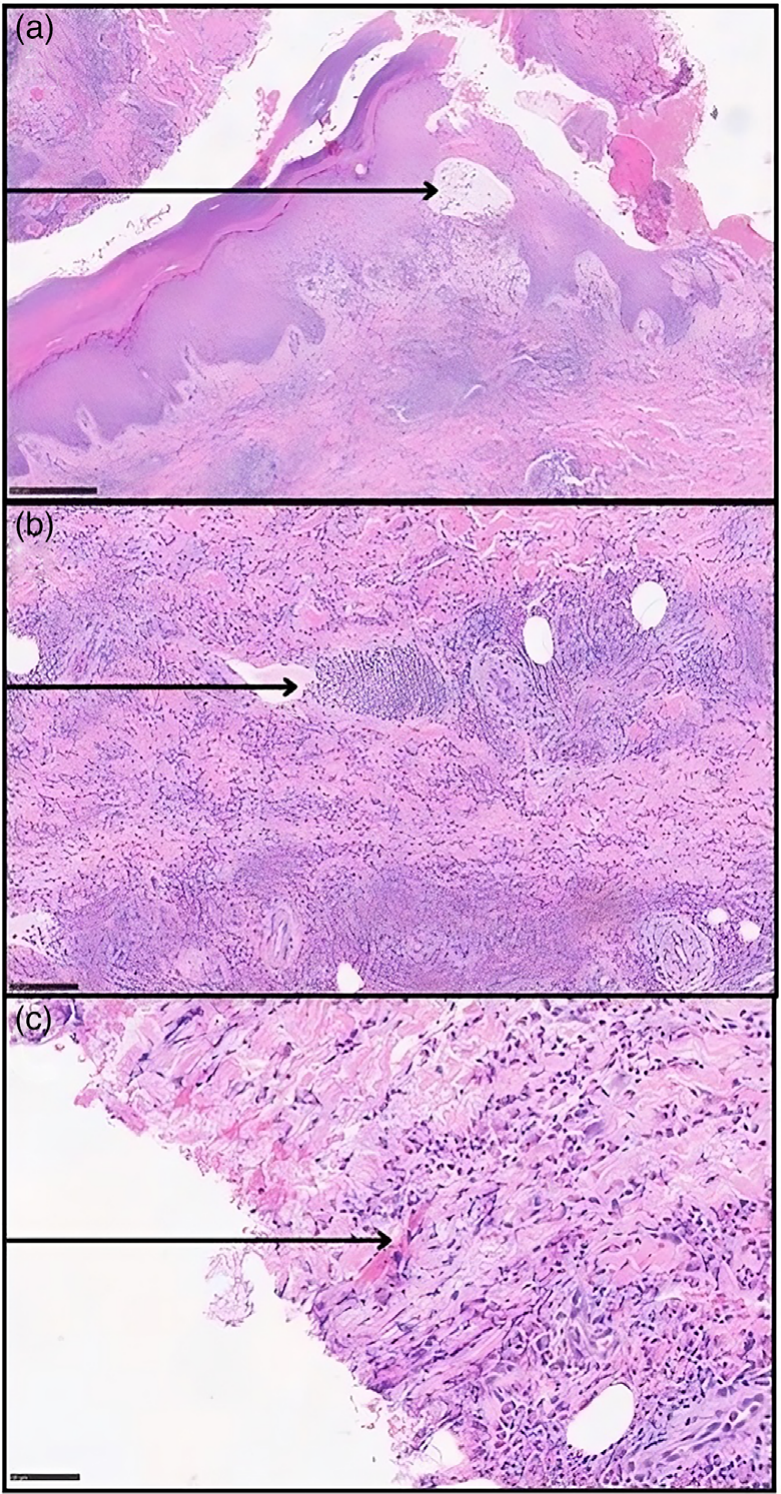
(a) Eosinophile Spongiose, gekennzeichnet durch intraepidermales Ödem (Pfeil) mit eosinophiler Infiltration zwischen Keratinozyten (Hämatoxylin‐Eosin‐Färbung [HE], Originalvergrößerung × 4). (b) Diffuses entzündliches Infiltrat, das hauptsächlich aus Eosinophilen, Lymphozyten und einigen mononukleären Zellen in der Dermis besteht; der Pfeil zeigt eine Ansammlung von Eosinophilen (HE, × 10). (c) Beispiel (Pfeil) einer „Flammenfigur“, einer amorphen eosinophilen Struktur, die aus proteinhaltigem Material und Zelltrümmern besteht und von degenerierten Eosinophilen und Histiozyten umgeben ist (HE, × 20).

Aufgrund der klinischen Merkmale, der Laboruntersuchungen und der histologischen Untersuchung wurde die Diagnose BWS gestellt. Die Patientin begann eine Therapie mit dem monoklonalen Anti‐IgE‐Antikörper Omalizumab (Xolair^®^, Novartis) in einer Dosierung von 300 mg subkutan alle vier Wochen und erholte sich innerhalb eines Monats vollständig (Abbildung 1d). Der Gesamt‐IgE‐Spiegel normalisierte sich allmählich wieder. Nach einem Jahr wurde die Behandlung abgebrochen, aber die Krankheit trat kurz darauf erneut auf. Die Behandlung mit Omalizumab wurde wieder aufgenommen, und die Wirksamkeit stellte sich umgehend wieder ein. Die Patientin erhält seit drei Jahren erfolgreich eine Anti‐IgE‐Therapie ohne bekannte Nebenwirkungen. Eine schriftliche Einverständniserklärung zur Veröffentlichung wurde eingeholt.

Ob WS eine eigenständige Erkrankung oder eine eosinophile Reaktion auf verschiedene Erreger ist, wird noch diskutiert.^1^, ^2^ Die Schwierigkeit bei der Diagnose dieser Erkrankung liegt in ihrer Seltenheit und ihrer Ähnlichkeit mit autoimmunen, infektiösen oder medikamenteninduzierten Dermatosen. Bei Verdacht auf BWS sollten mehrere Differentialdiagnosen in Betracht gezogen werden: bullöses Pemphigoid, bullöses Erysipel, bullöse Krätze, bullöse Arzneimittelreaktion mit Eosinophilie und systemischen Symptomen, Sweet‐Syndrom und andere.^1^, ^2^, ^3^, ^4^ Die Korrelation zwischen klinischen, pathologischen und labortechnischen Merkmalen ist für die richtige Diagnose von entscheidender Bedeutung.^5^

Obwohl es keine standardisierte Behandlung für WS gibt, stellen systemische Kortikosteroide die Erstlinientherapie dar, oft in Kombination mit steroidsparenden Wirkstoffen wie Dapson oder Cyclophosphamid bei rezidivierenden Erkrankungen.^1^, ^2^

Unsere Patientin ist bemerkenswert, da sie der erste berichtete Fall von BWS ist, der erfolgreich mit einem Anti‐IgE‐Antikörper behandelt wurde und eine langfristige Remission erreichte. In der Literatur wurden zwar einige WS‐Fälle beschrieben, die auf Omalizumab ansprachen, jedoch betraf keiner davon Patienten mit der bullösen Variante.^6^, ^7^, ^8^ Wir vermuten, dass unsere Patientin zunächst an CSU litt und anschließend ein BWS entwickelte, wobei beide Erkrankungen bekanntermaßen auf Omalizumab ansprechen. Obwohl selten, wurden Fälle von gleichzeitigem Auftreten von CSU und klassischem WS dokumentiert.^6^ Diese Assoziation sollte immer in Betracht gezogen werden, wenn Quaddeln gleichzeitig mit lang anhaltenden, juckenden und schmerzhaften Plaques auftreten.

Omalizumab ist eine etablierte Zweitlinienbehandlung der CSU, die empfohlen wird, wenn die Therapie mit Antihistaminika der zweiten Generation unwirksam ist. In der Pathophysiologie der CSU spielen Hautmastzellen eine zentrale Rolle als Effektorzellen: Ihre Aktivierung, Degranulation und Mediatorfreisetzung wird durch die Bindung von IgE an hochaffine FcεRI‐Rezeptoren ausgelöst, die auf der Oberfläche von Mastzellen und Basophilen exprimiert werden. Anti‐IgE‐Behandlungen reduzieren die Krankheitsaktivität deutlich, indem sie freies IgE binden, die FcεRI‐Expression auf Mastzellen und Basophilen herunterregulieren und so deren Aktivierung verhindern. Mehrere Studien haben gezeigt, dass FcεRI auf hautinfiltrierenden Eosinophilen bei atopischer Dermatitis und bullösem Pemphigoid hochreguliert ist,^10^ was den erfolgreichen Einsatz von Omalizumab bei diesen eosinophilen Erkrankungen erklären könnte. In ähnlicher Weise kann spekuliert werden, dass dermale Eosinophile bei WS FcεRI hochregulieren, das anschließend durch Omalizumab herunterreguliert wird, was zu klinischer Remission führt.^9^

Zusammenfassend zielt dieser Fallbericht darauf ab, das Bewusstsein für BWS und die verfügbaren Behandlungsmöglichkeiten zu schärfen. Omalizumab scheint eine vielversprechende therapeutische Strategie zu sein.

## DANKSAGUNG

Open access publishing facilitated by Universita degli Studi di Foggia, as part of the Wiley ‐ CRUI‐CARE agreement.

## INTERESSENKONFLIKT

Keiner.

## References

[1] Marzano AV , Genovese G . Eosinophilic dermatoses: Recognition and management. Am J Clin Dermatol. 2020;21(4):525‐539. 32394361 10.1007/s40257-020-00520-4

[2] Caputo R , Marzano AV , Vezzoli P , Lunardon L . Wells syndrome in adults and children: A report of 19 cases. Arch Dermatol. 2006;142(9):1157‐1161. 16983003 10.1001/archderm.142.9.1157

[3] Drago F , Ciccarese G , Merlo G , et al. Oral and cutaneous manifestations of viral and bacterial infections: Not only COVID‐19 disease. Clin Dermatol. 2021;39(3):384‐404. 34517997 10.1016/j.clindermatol.2021.01.021 PMC7849469

[4] Drago F , Ciccarese G , Agnoletti AF , et al. Neuro sweet syndrome: a systematic review. A rare complication of Sweet syndrome. Acta Neurol Belg. 2017;117(1):33‐42. 27659797 10.1007/s13760-016-0695-1

[5] Guglielmo A , Filippi F , Pileri A , et al. Bullous Wells Syndrome: A needle in the haystack. Int J Dermatol. 2021;60(4):e150‐e153. 33259051 10.1111/ijd.15250

[6] Ogueta I , Spertino J , Deza G , et al. Wells syndrome and chronic spontaneous urticaria: report of four cases successfully treated with omalizumab. J Eur Acad Dermatol Venereol. 2019;33(10):e388‐e391. 31106467 10.1111/jdv.15683

[7] Coattrenec Y , Ibrahim Yasmine L , Harr T , et al. Long‐term remission of Wells syndrome with omalizumab. J Investig Allergol Clin Immunol. 2020;30(1):58‐59. 10.18176/jiaci.0436 31530507

[8] Egeland Ø , Balieva F , Undersrud E . Wells syndrome: A case of successful treatment with omalizumab. Int J Dermatol. 2018;57(8):994‐995. 29707773 10.1111/ijd.14006

[9] Altrichter S , Fok JS , Jiao Q , et al. Total IgE as a marker for chronic spontaneous urticaria. Allergy Asthma Immunol Res. 2021;13(2):206‐218. 33474856 10.4168/aair.2021.13.2.206 PMC7840871

[10] Messingham KN , Holahan HM , Frydman AS , et al. Human eosinophils express the high affinity IgE receptor, FcεRI, in bullous pemphigoid. PLoS One. 2014;9(9):e107725. 25255430 10.1371/journal.pone.0107725 PMC4177878

